# Intent to Die and Suicide Attempts: Exploring Clinical and Circumstantial Correlates

**DOI:** 10.1192/j.eurpsy.2025.2358

**Published:** 2025-08-26

**Authors:** K.-U. Lee, J. T. Park, K. H. Choi

**Affiliations:** 1 Psychiatry; 2 Emergency medicine, Uijeongbu St. Mary’s Hospital, The Catholic University of Korea, Seoul, Korea, Republic Of

## Abstract

**Introduction:**

Intent to die is a crucial factor in assessing the risk of suicide attempts, which is difficult to measure.

**Objectives:**

This study examined the characteristics of suicide attempters based on intent to die and aimed to investigate whether clinical and circumstantial evidence can support the assessment of the intent to die.

**Methods:**

A total of 3486 suicide attempters who visited emergency department were divided into two groups: intent to die(n=1085, 31.1%) and no intent to die(n=2401, 68.9%). Demographic variables, clinical characteristics, and factors related to suicide attempt between the two groups were analyzed.

**Results:**

Suicide attempters who reported an intent to die were older (46.7 ± 21.7 vs. 40.5 ± 19.3, t=8.460, p < 0.001), had a higher proportion of males (41.1% vs. 33.4%, χ2=19.174, p < 0.001), were more likely to be unemployed (60.6% vs. 51.0%, χ2=26.954, p < 0.001), had lower socioeconomic status (34.1% vs. 23.4%, χ2=44.365, p < 0.001), and experienced more severe depression (76.6% vs. 49.6%, χ2=230.442, p < 0.001), intense emotions (92.9% vs. 80.0%, χ2=91.138, p < 0.001), agitation (45.5% vs. 40.4%, χ2=7.734, p < 0.01), and hopelessness/helplessness (86.1% vs. 60.7%, χ2=221.980, p < 0.001) compared to those who did not report an intent to die. Moreover, suicide attempters who reported an intent to die showed more repetitive/intense/continuous suicide ideation (79.8% vs. 42.8%, χ2=410.830, p < 0.001), a higher rate of multiple attempts (46.5% vs. 41.1%, χ2=8.637, p < 0.005), higher medical risk of death (3.6 ± 1.3 vs. 3.0 ± 1.1, t=15.633, p < 0.001), a higher total risk score (9.5 ± 2.2 vs. 8.3 ± 2.0, t=25.596, p < 0.001), and a lower total rescue score (12.1 ± 2.0 vs. 12.7 ± 1.9, t=8.649, p < 0.001) compared to those who did not report an intent to die. Some circumstantial factors such as planned attempts (19.4% vs. 2.3%, χ2=307.079, p < 0.001), presence of suicide notes (21.3% vs. 9.8%, χ2=83.625, p < 0.001), absence of regret (75.1% vs. 51.2%, χ2=174.849, p < 0.001), and high lethality of suicide methods (18.0% vs. 9.2%, χ2=56.161, p < 0.001) showed statistical significant differences, but some proportions of suicide attempters who did not report an intent to die also exhibited these circumstantial factors.

**Conclusions:**

The present study suggests that suicide attempters who reported an intent to die tend to have more severe psychopathologies and serious suicide attempts related to direct factors than suicide attempters who did not report an intent to die.

**Disclosure of Interest:**

None Declared

